# Food Insecurity and Chronic Disease Among Native Hawaiians and Pacific Islanders on the Continent During COVID-19

**DOI:** 10.1007/s40615-025-02638-9

**Published:** 2025-09-27

**Authors:** Marla Alohilani Tam-Hoy Barhoum, Maddy Rantala, Meghan Lindell, Pearl McElfish, Eric Lofgren, Denise Dillard, Erin Renee Morgan

**Affiliations:** 1 Institute for Research and Education to Advance Community Health, Elson S. Floyd College of Medicine, Washington State University, Spokane, WA USA; 2 Institute for Community Health Innovation, University of Arkansas for the Medical Sciences, Fayetteville, AR USA; 3 Paul G. Allen School for Global Health, College of Veterinary Medicine, Washington State University, Pullman, WA USA

**Keywords:** NH/PI: Native Hawaiians/Pacific Islanders (NH/PI); the use of the forward slash differentiates Pacific peoples and Native Hawaiians,, Health equity,, Food insecurity,, Nutrition,, Epidemiology

## Abstract

Financial precarity and food insecurity are strongly associated with poor health outcomes. Recently, the COVID-19 pandemic caused major disruptions to people’s livelihoods as well as interrupting supply chains, furthering economic hardship and exacerbating existing inequities. Native Hawaiian and Pacific Islander individuals, particularly those living on the continent, were especially impacted by the pandemic. This analysis used data from the Moana: Alternative Surveillance of COVID-19 in a Unique Population study to assess the economic and health correlates of food insecurity. We used the baseline survey data from 295 adult respondents and applied logistic regression to calculate adjusted Odds Ratios (aORs) and 95% Confidence Intervals (CIs). We used three definitions of food insecurity, including two definitions from the US Department of Agriculture and a third question that queried about food insecurity since the onset of the pandemic. Over half of participants expressed concerns around food security (56.0% worried about food, 52.5% reported food didn’t last). Experiencing a reduction in hours due to COVID-19 was positively associated with all measures of food insecurity, while being an essential worker was protective. We also found that people with diagnosed diabetes had higher odds of reporting that food did not last (aOR: 2.12; 95% CI: 1.05–4.41) or experiencing food insecurity since the beginning of the COVID-19 pandemic (aOR: 2.36; 95% CI: 1.19–4.72). Our findings support the growing body of literature indicating a relationship between food insecurity and cardiometabolic diseases. It also suggests that NH/PI on the continent may benefit from additional, culturally tailored interventions.

## Introduction

Poverty, food insecurity and poor nutrition independently and collectively contribute to negative health outcomes and disparities. Factors that influence spendable income, such as losing a job, having hours or wages reduced or losing access to government food support programs can have major consequences on a household’s financial situation. Furthermore, adding members to the household, bringing home a new baby, or establishing a multigenerational home can also alter a family’s financial wellbeing, and are frequently correlated with other social determinants of health (SDOH). The socioeconomic and physical environments in which we are born, live, learn, work, and age are often linked to health outcomes, whether positive or negative [1, 2]. When income is reduced, access to food can become uncertain and nutrition can be impacted [3]. Food insecurity is one of the nation’s top health and nutrition challenges, with 18 million households being food insecure in 2023 [4]. 

The United States Department of Agriculture (USDA) defines a household as food insecure if food consumption of one or more family members in the same household is reduced; eating patterns are irregular due to lack of money and other resources, or individuals face the inability to afford consistent meals, which leads to skipped meals, or eating smaller portions [5, 6]. Food insecurity and reduced access to nourishing foods are often associated with poor health outcomes, such as hypertension, diabetes, depression and other mental health challenges, poor sleep outcomes and overall lower general health standards [7, 8].

Increased food insecurity was one of the many economic consequences of COVID-19. For many households in the United States, mitigation efforts in the form of social distancing or stay at home orders had direct impact on people’s livelihoods. There were also macroeconomic forces stemming from large-scale issues in production and delivery chains, as well as the overall uncertainty felt by everyone from average consumers to investors [9, 10]. The increased food insecurity during COVID-19 and was higher among racial and ethnic minorities, households with children, and families where someone had their hours reduced or lost employment. Upwards of 54 million American households felt the impact of food insecurity during COVID-19, primarily during 2020 to early 2021 [11, 12]. In addition to the vulnerability caused by food insecurity, Native Hawaiians and Pacific Islanders (NH/PI) were highly vulnerable to COVID-19 and had elevated risk factors for infection, hospitalization, and death [13]. In the few states that reported disaggregated data, COVID-19 incidence among the NH/PI community was higher when compared to Whites and other minority groups [14–19]. This is attributed to NH/PI having higher rates of pre-existing chronic diseases, making this group more susceptible to illness during a public health crisis like the COVID-19 outbreak [20, 21].

According to the 2020 Census, 47% of Native Hawaiians in the United States live in Hawaiʻi and 53% reside on the continent. For the first time, data confirms this geographic shift and displacement of this indigenous population. In addition to the growing Native Hawaiian population in the continental United States, migration from Marshall Islands and those from Pasifika has also vastly increased. While Polynesian, Micronesian, and Melanesian cultures are distinct, one shared experience across these groups is the movement away from their indigenous homelands known as diaspora [22]. In all three island regions, NH/PIs are being forced to relocate due to rising costs of living, however living away from traditional homelands can have severe negative impacts on individuals, families, and communities [23].

Despite many relocating to the US continent for a better life, over 1/3 of NH/PIs nationwide report being food insecure [24]. Recent disaggregated data shows 75.5% of Marshallese households experience food insecurity, the highest of any racial or ethnic group [25]. Even prior to the COVID-19 pandemic, an estimated 33.8% of NH/PI households utilized SNAP (Supplemental Nutritional Assistance Program, commonly known as food stamps), another indicator that this population faces heightened levels of food insecurity [26].

Although there is well-documented variation in personal and lived experiences, most of the research on health and health disparities experienced by NH/PIs has primarily focused on those living in Hawai’i or across the Pacific Islands. To better understand this understudied and rapidly growing population, we partnered with two NH/PI community organizations to measure the impact of the COVID-19 pandemic on NH/PI communities. We explored the impact of COVID-19 on food insecurity among NH/PI adults living on the continent. Additionally, we measured the relationship between food insecurity and chronic health conditions among this unique population. Our team hopes the findings from this study can heighten the awareness of the importance of culturally relevant resources and health-based interventions to NH/PI people living on the continent.

## Methods

The Moana: Alternative Surveillance of COVID-19 in a Unique Populations (MASC-UP) study was designed to determine the impact of the COVID-19 pandemic on NH/PI adults living in the continental US (i.e., on the continent). We partnered with two community-based organizations that serve Pacific Islanders on the continent: Pacific Islander Community Association of Washington (PICA-WA) in Federal Way, Washington, and the Center for Pacific Islander Health (CPIH) in Springdale, Arkansas. There was an additional remote arm of the study which recruited participants via the internet, largely through social media advertising, and in-person cultural events. Participation in MASC-UP entailed the same study procedures and eligibility criteria across all study sites, including the remote arm of the study, which was implemented by Washington State University staff based in Seattle, Washington.

The MASC-UP study was designed as participatory surveillance program for COVID-19. To better understand the COVID-19 pandemic among NH/PI adults, we asked participants to complete a baseline survey and contact diary. Participants were then asked to take daily temperature readings using a Bluetooth enabled thermometer and complete a brief monthly survey which covered symptoms of COVID-19. Additional details of the MASC-UP methodology have been previously described elsewhere [27]. Most critical to these analyses, all participants were asked to complete surveys at baseline and follow-up points (12 months for those recruited from PICA-WA or 6-months for those recruited at CPIH or online). Our original protocol intended to follow up with participants for 12 months, however, after launching the first site, participant retention became a notable problem. To address this, we amended our protocol when we launched the CPIH and online arms of the study such that it only required 6 months of follow-up. Despite the change in follow-up duration, retention remained lower than anticipated; thus, this paper focuses on data from the baseline survey to reduce biasing results by utilizing only data from individuals who were able to complete follow-up data collection.

### Measures

We collected demographic data from participants, including age, NH/PI ethnic identity (Micronesian, Melanesian, or Polynesian), employment status (permanent employment, temporary employment, or unemployed), household composition (living alone, living with a partner or other individuals of the same generation, living with children, living with 3 + generations, or another living arrangement), and ZIP code. We also asked if they had been employed as an essential worker during the pandemic, if they faced a reduction in hours or wages since the onset of the pandemic, and if they had lost a job due to COVID-19 or related mitigation efforts.

In addition to demographics, participants were asked about food security. Using questions developed by the United States Department of Agriculture, participants were asked the extent they agreed with the following statements: “Within the past 30 days, we worried whether our food would run out before we got money to buy more,” and “Within the past 30 days, the food we bought just didn't last and we didn't have money to get more.” Response options were included: “Never true,” “Sometimes true,” or “Always true.” For the analysis, we combined responses that indicated the participant had experienced any amount of food insecurity, i.e., any option other than “Never true” [4, 28]. In addition, we assessed how access to food was impacted by COVID-19 or the related mitigation efforts. The baseline survey question asked, “Since the beginning of the pandemic, have you had difficulty getting enough food?” Response options were yes or no.

We also assessed specific chronic health conditions. Participants were asked if a physician or other healthcare professional had ever diagnosed them with asthma, hypertension, or diabetes. They were also asked if they had been diagnosed with or tested positive for COVID-19.

### Analysis

To focus on NH/PI adults on the continent, we excluded participants who reported living in Hawai`i or American Samoa and excluded individuals who did not provide a ZIP code. We first tabulated descriptive statistics for demographic characteristics. We then used logistic regression to calculate odds ratios (ORs) and 95% confidence intervals (95% CIs). We used ORs to measure the relationship between economic factors and food insecurity outcomes. We subsequently used ORs to measure the relationship between food insecurity and health conditions. We then adjusted models for sex, age, essential worker status, and reporting reduced wages/hours due to COVID-19. All analyses were performed in R version 4.4.0. The MASC-UP study was reviewed and approved by Washington State University and University of Arkansas for the Medical Sciences Institutional Review Boards.

## Results

Across the three sites, 339 participants completed the baseline survey and 295 lived on the continent. Demographic characteristics can be found in Table 1. The majority of study participants identified as female (68.6%). Most participants identified as either Polynesian (45.1%) or Micronesian (44.4%). Over half expressed concerns around food security (56.0% worried about food, 52.5% reported food didn’t last).

**Table 1:** Sociodemographic characteristics of NH/PI adults participating in the MASC-UP study

	N	%
NH/PI Ethnicity*		
Micronesian	131	44.4%
Melanesian	27	9.2%
Polynesian	133	45.1%
Sex/Gender		
Male	84	28.7%
Female	201	68.6%
Other	8	2.7%
Age		
18–24	51	17.6%
25–34	65	22.5%
35–44	59	20.4%
45–54	51	17.6%
55–64	36	12.5%
65 +	27	9.3%
Food Insecurity*		
Worried About Food	153	56.0%
Food Didn't Last	138	52.5%
Food Since COVID	115	39.7%

*Categories are not mutually exclusive

When assessing economic predictors of food insecurity (Table 2), experiencing a reduction in hours or wages due to COVID-19 was positively associated with all three measures of food insecurity. We also found a positive association between unemployment and food insecurity, regardless of how it was defined. Conversely, being employed as an essential worker during the pandemic was negatively associated with food insecurity. Finally, we also found that living in multigenerational households was associated with increased odds of food insecurity, though, due to relatively small numbers, this estimate was not precise.

**Table 2:** Socioeconomic predictors of food insecurity among NH/PI adults living on the continent

		**Worried About Food**		**Food Doesn't Last**		**Food COVID**	
	*n* (%)	Crude OR	Adjusted OR*	Crude OR	Adjusted OR*	Crude OR	Adjusted OR*
Employed as Essential Worker	102 (35.1%)	0.44 (0.26–0.72)	0.47 (0.24–0.91)	0.51 (0.30–0.84)	0.61 (0.31–1.20)	0.70 (0.42–1.15)	0.71 (0.36–1.43)
Wages or Hours Reduced due to COVID	103 (35.3%)	1.75 (1.05–2.94)	2.57 (1.24–5.45)	1.70 (1.03–2.86)	2.86 (1.36–6.15)	3.23 (1.97–5.37)	4.47 (2.12–9.71)
Lost Job due to COVID	61 (20.9%)	1.99 (1.08–3.75)	1.22 (0.52–2.89)	2.10 (1.15–3.95)	1.32 (0.57–3.13)	4.55 (2.50–8.53)	2.29 (1.02–5.28)
Employment Status							
Permanent Employment	140 (48.6%)	1.00 (Ref)	1.00 (Ref)	1.00 (Ref)	1.00 (Ref)	1.00 (Ref)	1.00 (Ref)
Temporary Employment	25 (8.7%)	1.75 (0.73–4.33)	1.82 (0.68–5.06)	2.22 (0.93–5.52)	2.16 (0.78–6.19)	1.20 (0.47–2.91)	0.97 (0.30–2.94)
Unemployed	123 (42.7%)	2.92 (1.73–5.00)	2.80 (1.32–6.11)	3.47 (2.03–6.01)	3.48 (1.61–7.78)	1.73 (1.05–2.86)	2.12 (1.00–4.60)
Household Composition							
Living Alone	15 (5.1%)	1.00 (Ref)	1.00 (Ref)	1.00 (Ref)	1.00 (Ref)	1.00 (Ref)	1.00 (Ref)
Living w/Partner	58 (19.9%)	1.60 (0.50–5.72)	4.29 (0.99–23.72)	1.77 (0.52–7.10)	4.12 (0.91–23.71)	1.03 (0.32–3.69)	4.25 (0.80–28.10)
Living w/Children	147 (50.3%)	2.22 (0.75–7.46)	4.84 (1.21–25.28)	2.98 (0.96–11.19)	5.87 (1.40–31.90)	1.12 (0.38–3.75)	4.10 (0.84–25.31)
3 + Generations	51 (17.5%)	12.29 (3.40–51.04)	29.82 (6.25–183.22)	12.53 (3.47–54.34)	24.79 (5.17–152.03)	3.37 (1.04–12.23)	15.08 (2.84–100.98)
Other	21 (7.2%)	2.22 (0.56–9.57)	6.12 (1.14–40.48)	1.60 (0.37–7.57)	4.13 (0.71–28.49)	0.80 (0.19–3.46)	2.49 (0.37–19.02)

* Adjusted for sex, age, and present categories

Finally, we measured the association between food insecurity and chronic health conditions (Table 3). Nearly half of the study sample reported that they had either tested positive for COVID-19 or been diagnosed with COVID-19 by a healthcare provider (*n* = 130; 44.8%). Asthma and COVID-19 infection were both associated with roughly 50% decreased odds of worrying about food. Prior COVID-19 infection was also associated with a reduction in reporting that food didn’t last (aOR: 0.57; 95% CI: 0.33–0.99) or that food insecurity had been a concern since the beginning of the pandemic (aOR: 0.43; 95% CI: 0.24–0.75). A prior hypertension diagnosis and a prior diabetes diagnosis were both endorsed by about one-fifth of participants (21.2% and 20.9%, respectively). Not enough food was associated with an approximately two-fold increase in the odds a diagnosis of diabetes (aOR: 2.12; 95% CI: 1.05–4.41) as was reporting food insecurity since the beginning of the COVID-19 pandemic (aOR: 2.36; 95% CI: 1.19–4.72). There were no statistically significant associations between any measure of food insecurity and hypertension.

**Table 3:** Relationship between chronic health conditions and food insecurity among NH/PI adults living on the continent

		Worried About Food		Food Doesn't Last		Food COVID	
	*n* (%)	Crude OR	Adjusted OR*	Crude OR	Adjusted OR*	Crude OR	Adjusted OR*
Asthma	38 (13.1%)	0.48 (0.23–0.97)	0.38 (0.16–0.84)	0.53 (0.25–1.08)	0.42 (0.18–0.94)	0.90 (0.43–1.81)	0.56 (0.23–1.29)
Hypertension	62 (21.2%)	0.92 (0.51–1.65)	0.85 (0.41–1.75)	1.18 (0.65–2.17)	0.94 (0.44–2.01)	0.97 (0.54–1.72)	1.07 (0.51–2.21)
Diabetes	61 (20.9%)	1.75 (0.97–3.24)	1.69 (0.86–3.38)	2.50 (1.35–4.81)	2.12 (1.05–4.41)	1.91 (1.08–3.40)	2.36 (1.19–4.72)
COVID-19	130 (44.8%)	0.46 (0.28–0.75)	0.50 (0.29–0.86)	0.54 (0.33–0.88)	0.57 (0.33–0.99)	0.56 (0.34–0.90)	0.43 (0.24–0.75)

*Adjusted for sex, age, essential worker status, and a reduction in wages or hours due to COVID-19

## Discussion

MASC-UP identified that more than one third of participants lost jobs or had wages or hours reduced due to COVID and over half of MASC-UP participants reported at least one type of food insecurity. Food insecurity was related to diabetes as a nutrition-related chronic diseases. Our results are in line with other findings that the NH/PI population in the United States were disproportionately impacted by COVID-19 given the proportion who were infected with COVID-19 [29–31]. This population was not only affected by COVID-19 infections, hospitalizations, and mortality but were also highly impacted by financial ramifications of the pandemic, including food insecurity. During the pandemic, NH/PI communities were frequently faced with the decision to either work despite infection risk or struggle to feed their families.

Our study contributes to the growing body of literature supporting a relationship between not having enough food and diabetes [32]. Research in Hawai`i demonstrated that as NH/PI health declined, food insecurity within their households increased. Direct connections were made between reduced household income and increased risk factors for diabetes [33–35]. The findings in this study focused on NH/PIs living on the continent and directly aligned with other analyses that aimed to better understand how COVID-19 exacerbated health inequities.

MASC-UP participants who reported not having enough food or that food insecurity was a problem since the start of the pandemic had two times the odds of reporting that they had been diagnosed with diabetes compared to people who did not experience these types of food insecurity. They were more likely to report having diabetes or that diabetes was more common among the group that experienced food insecurity. Socioeconomic and environmental contributors have a complex relationship in how SDOH influence both health outcomes and disparities [36]. This is consistent with prior literature, which has shown that diagnosed diabetes is quantitatively linked to both poverty level and employment status, after adjusting for sex, race/ethnicity and age [37]. When we further adjusted for employment status and other economic variables, there was still a relationship between food insecurity and diabetes, indicating a causal mechanism that goes beyond the absence of economic resources.

The meaning of negative relationship between measures of food insecurity and COVID-19 or asthma diagnosis is less clear. It is plausibly the result of residual confounding. Despite adjusting for some economic variables (i.e., essential worker status and reduction of hours since the beginning of the COVID-19 pandemic), it is likely that there were additional, unmeasured economic factors that explain the relationship. For instance, individuals who worked in healthcare were more likely to be exposed to COVID-19 than individuals working in grocery; while both occupations were classed as essential workers, healthcare workers had a greater risk of exposure and greater earnings than retail employees. While our respondents were given an opportunity to indicate whether their occupation matched any of 11 different fields of work, most indicated that their specific field of employment was not listed as a response option, so we were not able to directly measure this phenomenon in our data. The high rate of “none of the above” type responses may be in part attributable to state-level differences in which occupations were considered essential and thus exempted from mitigation efforts.

### Limitations

While this study addressed many gaps in the literature, it is not without weaknesses. We relied on self-reported diagnoses of health conditions. Availability of healthcare services is highly associated with screening among adults, indicating that uninsured NH/PIs have higher rates of undiagnosed diabetes due to limited medical access [38, 39]. Due to being under- or uninsured, it is plausible that NH/PIs may have elevated rates of chronic diseases that have not been properly diagnosed, which would lead to underreporting in our study. However, those that are unable to visit a doctor on a regular basis due to costs are more likely to be experiencing food insecurity. It is likely that any misclassification due to diabetes going undiagnosed attenuates our results towards the null. This entangled relationship is most likely stronger than we observed, as food and economic insecurity spiral into all aspects of daily life.

Other potential limitations stem from our recruitment methodology. Community-based partnerships were essential to MASC-UP success via outreach, recruitment, participation, and retention. With the assistance of these two cultural-based organizations and their site staff, we were able to reach a higher number of participants at two sites on the US continent. Both CPIH and PICA sites recruited directly from their office locations. For our site in Arkansas, CPIH staff offer reduced-cost clinic services and frequently serve as interpreters for their Marshallese populations in medical settings; thus, it is possible that CPIH MASC-UP participants had more comorbidities than the underlying population. PICA staff also recruited participants from the population of individuals utilizing their other services such as assistance navigating various safety net programs, including SNAP/EBT (food stamps), housing assistance, or registering for Apple Care (the Washington State version of Medicaid expansion). Thus, participants from the Washington site may have been in a more financially precarious position than other NH/PI individuals in the community. While this may limit the extent to which our sample was representative of the larger NH/PI population on the continent, partnership with these community organizations was also a great strength of the study as language was not a barrier to participation. Our NH/PI community partners added layers of personalization and direct care, weaving in both cultural awareness and sensitivity.

### Cultural Considerations

Food insecurity and social determinants of health such as poverty must be considered in cultural, historical, sociopolitical, and demographic context [40]. As a direct impact of colonialism via western contact, NH/PIs continue to face systemic oppression. In addition, historical trauma can be passed down through generations, impacting both mental and physical health, and is often difficult to recover from. Scientists, clinicians, and NH/PI scholars and community health advocates have demonstrated that these high rates of health disparities and health inequities stem from SDOH, historical trauma, racial discrimination, and lifestyle changes [41, 42].

The underlying issue of food insecurity is a chain reaction of colonization, systemic oppression, and historical trauma which leads to prejudice, discrimination, and stigmatization that continues to impact NH/PI individuals today, including adverse health outcomes [43]. Health and well-being are a familial interwoven bond between NH/PIs and their *ʻāina* (land, sea, traditional fresh foods, natural environment) [44]. Aloha (love) for the ʻāina is deeply rooted and a key component to Hawaiian cosmology and ideology. Their lived experience on the continent is often very different than living at home in the islands, where they are disconnected from these cultural pillars: *ʻāina, ʻai pono* (traditional foods)*, ʻohana* (family), *ʻōlelo (*language), cultural customs and traditional belief systems that shape their personal identity [45]. The strong emphasis on connection to ʻāina, among other cultural elements, is one reason to believe that NH/PI living in diaspora may face unique challenges. By creating culturally relevant interventions—e.g., interventions that incorporate components of NH/PI tradition with evidence-based interventions—individuals living in diaspora are provided with a space in which they can share their experiences and build a community. Be it through facilitating community or by encouraging food sovereignty, creating opportunities for NH/PIs to reconnect with culture and traditional foods, regardless of geographic location, may be another opportunity to lessen the burden of food insecurity in this population.

Chronic health conditions are also likely to be one manifestation of intergenerational trauma among the NH/PI community. Researchers in Hawai`i analyzed the adapted historical loss scale among the Native Hawaiian community, paving the way for quantitative assessments of historical and intergenerational trauma and a better understanding of how these three factors of (1) loss of culture, (2) loss of people, and (3) cultural mistreatment is vital to understand the connection trauma as a determinant of health and to shift focus on a future of healing, health and well-being [46–49]. Culturally grounded interventions—programming that seeks to build novel interventions beginning from traditional cultural activities or rooted in community values—have yielded positive outcomes [50, 51]. In one study, the traditional Hawaiian dance of hula was used to test the health benefits of physical activity in relation to the management of hypertension in NH/PIs [52]. More preventative health interventions are needed that blend culture in this deep and meaningful way, such as those which utilize *ʻohana* (family-based) frameworks in an environment that can be sustainable in real-life [53]. Successful health study models are integral for understanding the health behaviors of NH/PIs when designing new health promotion and disease prevention programs. Having culturally connected community partners that specifically serve NH/PI populations helped to build trust with MASC-UP participants. Similarly, many of our MASC-UP staff members are NH/PI, which positively contributed to community engagement, recruitment, and retention.

### Implications for Research and Practice

As NH/PI populations continue to grow on the continent, there is a need to ensure that solutions to food insecurity don’t exacerbate existing chronic health conditions. Local food banks often lack access to fresh, healthy foods [54, 55]. Canned meats, high-sodium canned foods, sugar-sweetened beverages and cereals and ultra-processed and packaged foods may have a high shelf life but contain high contents of additives, preservatives, chemicals, and artificial ingredients [56]. Nutrition education is also an important factor [57]. Access to traditional foods programs could uplift food sovereignty and cultural preservation and help NH/PIs navigate away from processed Western foods. Even as life post-pandemic returns to normal, it is unclear how or if the economic precarity revealed by COVID-19 will be addressed by policymakers or revert to previous levels of security [9, 10]. Given the many other benefits to supporting food sovereignty, supporting this access is critical in addressing nutrition-related health equity regardless of the long-term consequences of pandemic mitigation efforts.

The MASC-UP study improves the visibility of NH/PIs in data and research [58–61]. Data disaggregation is imperative to advance health equity among the NH/PI population. In 2021, 71% of Native Hawaiians also identified with another race in the American Community Survey [62]. Due to the use of a “multiracial” category, over half of Native Hawaiians are made invisible on surveys or government forms. Data combining NH/PIs with other racial or ethnic groups causes both undercounting and misclassification at the expense of communities with the greatest needs [30, 62]. It is critical that data collection is done in a pono (ethical) manner, in which every racial and ethnic group is seen as distinctive and unique. This is particularly pertinent when considering the unique challenges faced by NH/PI living in diaspora. Health research focused on experiences of NH/PI must consider the potential differences for those living in diaspora. This research, and the solutions it suggests, must work to ensure that the solutions are accessible to all NH/PI individuals, even those rendered invisible by policies of data aggregation and living great distances away from their traditional homelands.

## Data Availability

Select subsets of the data may be provided to other investigators upon request. Please contact the senior/corresponding author.
